# Sex Differences in Cognitive Abilities Among Children With the Autosomal Dominant Alzheimer Disease Presenilin 1 E280A Variant From a Colombian Cohort

**DOI:** 10.1001/jamanetworkopen.2021.21697

**Published:** 2021-08-31

**Authors:** Joshua T. Fox-Fuller, Arabiye Artola, Kewei Chen, Margaret Pulsifer, Dora Ramirez, Natalia Londono, Daniel C. Aguirre-Acevedo, Clara Vila-Castelar, Ana Baena, Jairo Martinez, Joseph F. Arboleda-Velasquez, Jessica B. Langbaum, Pierre N. Tariot, Eric M. Reiman, Francisco Lopera, Yakeel T. Quiroz

**Affiliations:** 1 Department of Psychiatry, Massachusetts General Hospital, Harvard Medical School, Boston; 2 Department of Psychological and Brain Sciences, Boston University, Boston, Massachusetts; 3 Department of Applied Psychology, Bouvé College of Health Sciences Northeastern University, Boston, Massachusetts; 4 Banner Alzheimer's Institute, Phoenix, Arizona; 5 School of Mathematical and Statistical Sciences, Arizona State University, Tempe; 6 Department of Neurology, College of Medicine-Phoenix, University of Arizona, Tempe; 7 Grupo de Neurociencias, Universidad de Antioquia, Medellín, Antioquia, Colombia; 8 Schepens Eye Research Institute of Massachusetts Eye and Ear, Harvard Medical School, Boston; 9 Massachusetts Eye and Ear, Harvard Medical School, Boston; 10 Athinoula A. Martinos Center for Biomedical Imaging, Charlestown, Massachusetts; 11 Department of Neurology, Massachusetts General Hospital, Boston

## Abstract

**Importance:**

We previously reported that children with the autosomal dominant Alzheimer disease (ADAD) presenilin 1 (*PSEN1*) E280A variant had early life plasma biomarker findings consistent with amyloid β overproduction. However, the cognitive functioning of children with this variant has not been characterized vs those without the variant.

**Objective:**

To test whether cognitive functioning of children with and without the *PSEN1* E280A variant in the same ADAD cohort differed by genetic status (ie, *PSEN1* variant) and sex.

**Design, Setting, and Participants:**

This cohort study was conducted among 1354 children (including 265 children with the variant) aged 6 to 16 years recruited from the Alzheimer Prevention Initiative Colombia Registry. Participants from the city of Medellín and surrounding suburban areas traveled to the University of Antioquia to undergo all procedures. Participants were administered a Spanish version of the *Wechsler Intelligence Scale for Children, Fourth Edition (WISC-IV)* to measure general cognitive functioning. Data were analyzed from July through November 2020.

**Main Outcomes and Measures:**

Univariate general linear models were used to characterize differences on *WISC-IV* cognitive performance by genetic status, sex, and the interaction of genetic status with sex. Urbanity, socioeconomic status, and education were entered as covariates.

**Results:**

Among 1354 children with ADAD (695 [51.3%] girls; mean [SD] age, 11.64 [2.64] years), there were 265 children with the variant (19.6%) and 1089 children without the variant (80.4%). Children with and without the variant did not differ by demographic variables or performance on *WISC-IV* indices. Irrespective of genetic status, boys had statistically significantly decreased mean scores on indices for working memory (90.27 [95% CI, 89.21-91.34] vs 92.99 [95% CI, 91.98-93.99]; mean difference = −2.72; *P* < .001), perceptual reasoning (91.56 [95% CI, 90.47-92.65] vs. 93.27 [95% CI, 91.23-94.30]; mean difference = −1.71; *P* = .03), and verbal comprehension (88.69 [95% CI, 87.54-89.84] vs. 90.81 [95% CI, 89.73-91.90]; mean difference = −2.12; *P* = .009) compared with girls. In the interaction between sex and genetic status, boys with the variant had worse mean working memory index performance (88.78 [95% CI, 86.86-90.70]) than girls with the variant (93.75 [95% CI, 91.95-95.55]; mean difference = −4.97; *P* = .001), as well as boys (91.77 [95% CI, 90.85-92.70]; mean difference = −2.99; *P* = .04) and girls (92.22 [95% CI, 91.32-93.13]; mean difference = −3.44; *P* = .009) without the variant.

**Conclusions and Relevance:**

This study found that boys with the *PSEN1* variant had decreased working memory abilities compared with girls with the variant and boys and girls without the variant, suggesting a sex-specific genetic risk in early life cognitive performance among individuals with the *PSEN1* variant. This increased risk of future cognitive difficulties among boys with the variant may have important downstream implications for learning and academic achievement and could be associated with sex differences seen in adulthood on episodic memory measures.

## Key Points

**Question:**

Do children from the Colombian kindred with autosomal dominant Alzheimer disease (ADAD) differ in general cognitive functioning based on sex or genetic status?

**Findings:**

In this cohort study of 1354 Colombian children (including 265 children with the presenilin 1 E280A variant), those with and without the variant did not differ on cognitive abilities. However, boys with the variant exhibited statistically significantly worse working memory performance compared with girls with the variant, as well as boys and girls without the variant.

**Meaning:**

This study found that boys with the ADAD variant had increased risk of worse cognitive performance compared with girls with and without the variant, as well as boys without the variant.

## Teaser

This cohort study investigates differences in cognitive functioning by sex and genetic variant status among Colombian children the autosomal dominant Alzheimer disease presenilin 1 E280A variant.

## Introduction

Individuals with autosomal dominant Alzheimer Disease (ADAD) variants develop early onset Alzheimer disease (AD) with nearly 100% penetrance, providing a unique opportunity to characterize the earliest biological and cognitive changes associated with the disease.^1^ Biomarker investigations of families with ADAD variants have revealed AD-related brain changes prior to the onset of clinical symptoms.^2^ We previously reported that children with the presenilin 1 (*PSEN1*) E280A ADAD variant, compared with child-aged family members without this variant, exhibited plasma biomarker levels consistent with early life amyloid β overproduction, as well as increased gray matter volume in temporal regions and decreased functional magnetic resonance imaging deactivation of posterior parietal regions during a memory encoding task.^3^ These AD-related biomarker differences were seen approximately 3 decades before the median age of onset of mild cognitive impairment (MCI) in individuals with the *PSEN1* E280A variant.^2^ We also recently reported that women with the *PSEN1* E280A variant who were not cognitively impaired had better verbal episodic memory performance compared with men with the variant who were not impaired,^4^ consistent with research that has found greater performance on verbal memory tests among healthy older women compared with healthy older men^5^ (although women with MCI or dementia may have more precipitous cognitive decline compared with men with cognitive impairment^6^). To our knowledge, however, no study has investigated the cognitive performance of children with ADAD variants compared with those without variants, nor the possible sex differences in cognitive performance in childhood in this population.

In this study, we used a neuropsychological measure, a Spanish version of the *Wechsler Intelligence Scale for Children, Fourth Edition *(*WISC-IV*),^7,8^ to assess cognitive functioning among children with the *PSEN1* E280A variant and age-matched family members without the variant from the Colombian ADAD kindred. The literature on sex differences in cognition among children without developmental disorders is variable, with boys and girls showing distinct patterns of working memory strengths.^9^ However, previous research on the cognitive functioning of adult members from this *PSEN1* kindred^1,10^ and findings from the literature about cognitive performance in children with the apolipoprotein ε4 (*APOE* ε4) variant,^11,12,13^ a known genetic risk factor associated with the more common sporadic form of late-onset AD, suggest that there may be differences in cognitive performance among children from this kindred. We hypothesized that children with an ADAD variant would perform worse on tests of verbal intellectual abilities compared with children from the same family without a variant and that boys with a variant would perform worse than girls with a variant across all *WISC-IV* indices.^4^

## Methods

This cohort study was conducted under guidelines approved by local institutional review boards. Ethics approval was obtained from the University of Antioquia Ethics Committee in Colombia. Parents or guardians of the participants provided written informed consent, and children assented before enrollment in the study and completion of the procedures. All data were acquired by investigators who were masked to the participants’ genetic status. This study followed the Strengthening the Reporting of Observational Studies in Epidemiology (STROBE) reporting guideline.

### Study Design, Setting, and Participants

A total of 1499 children were recruited from the Alzheimer Prevention Initiative Colombia Registry, which currently includes more than 6000 living members of the *PSEN1* E280A kindred, approximately 1200 of whom have the variant.^1^ Individuals with this variant have a median age of onset of 44 years (95% CI, 43 years-45 years) for MCI and 49 years (95% CI, 49 years-50 years) for dementia.^2^ Given that *PSEN1* E280A is an autosomal dominant variant, all children with the variant were heterozygous for *PSEN1* E280A and had a biological parent who also had the variant.^1^ A blood sample was taken to confirm genetic status.

All children in the recruited sample lived within a 100-mile radius of Medellín, Colombia. Participants were asked to identity their sex assigned at birth, using a binary definition of sex (ie, male or female). Potential participants were screened and excluded in advance for the presence of neurological disorders (eg, developmental disabilities, learning disorders, history of traumatic brain injury, and epilepsy) and chronic psychiatric disorders (eg, history of psychiatric hospitalizations, schizophrenia, and substance abuse disorders).

Participants were administered a Spanish version of the *WISC-IV*, a measure of cognitive functioning in children.^7,8^ Of 1499 children in the database, 145 participants with a Full Scale Intelligence Quotient (FSIQ) score lower than 70 on the *WISC-IV* (including 16 children with the variant) were excluded from the study, given that an FSIQ less than 70 is suggestive of an intellectual disability.^8^ We included 1354 children in the analysis, including 265 individuals (19.6%) with the *PSEN1* E280A variant.

Participants in this study were aged 6 to 16 years (estimated years until onset of MCI [EYO], −38 to −28 years) and belonged to the same extended families who descended from a shared ancestor with the *PSEN1* E280A variant. There were 457 participants (including all 265 children with the variant) who had a biological parent with the variant, whereas 897 children without the variant had an extended nonparent relative with the variant (eg, an uncle or aunt or a grandparent). The 192 children without the variant who had a biological parent with the variant were considered in a post hoc subsample analysis. This was done to determine if performance comparisons on the *WISC-IV* differed when comparing children with the variant against individuals without the variant who had a parent with the variant vs when comparing against the entire larger sample of children without the variant.

### Cognitive Measure

Data were acquired at the University of Antioquia in Medellín, Colombia. Cognitive tests were administered in Spanish by psychologists trained in neuropsychological assessment (DR and NL). The *WISC-IV* is a neuropsychological assessment commonly used to measure cognitive abilities in children aged 6 to 16 years.^8^ The *WISC-IV* has 15 subtests, although the first 10 subtests are considered the core subtests and yield an FSIQ and the scores on the following 4 indices: verbal comprehension index (VCI), perceptual reasoning index (PRI), working memory index (WMI), and processing speed index (PSI).^8^ The VCI consists of 3 subtests: vocabulary, similarities, and comprehension. The VCI is used to measure a child’s language-based intellectual abilities. The PRI measures nonverbal fluid reasoning and is assessed with the following subtests: matrix reasoning, picture concepts, and block design. The WMI evaluates attention and concentration and consists of 2 subtests: digit span and letter-number sequencing. The PSI measures the speed of mental processing^14^ and contains 2 timed subtests: coding and symbol search. Scores on the *WISC-IV* in this study were normed relative to a sample of Spanish-speaking children and adolescents from Mexico, given that the *WISC-IV* had not been normed in a Colombian population at the time of this report. This Spanish *WISC-IV* version is referred to as the *Escala Wechsler de Inteligencia para Niños-IV*.^7,8^ Raw *WISC-IV* data from the subtests were normed using the participant’s age and transformed into scaled scores, which have a mean (SD) of 10 (3). The *WISC-IV* technical manual^8^ explains in detail how the subtests are converted into the 4 index scores, which have a mean (SD) of 100 (15).

### Statistical Analysis

#### Demographic Variables

Potential differences in age and education between children with and without the variant (as well as between boys and girls with the variant) were examined using Welch 2 independent sample *t* tests. We used χ^2^ tests to examine potential differences between children with and without the variant in sex ratio, current living zone (ie, urban vs rural), and socioeconomic status (SES; defined in Colombia by 6 strata: lower low, low, upper low, medium, medium high, and high).

#### Cognitive Variables: *WISC-IV*

Univariate general linear models were used to characterize the association between genetic status and performance on the VCI, WMI, PRI, and PSI; these models were run on SPSS statistical software version 26 (IBM). For each dependent variable (ie, each *WISC-IV* index score), we examined if there were differences by genetic status (model 1), sex (model 2), and the interaction of genetic status with sex (model 3). Education, SES, and urbanity were entered as covariates in all models, and Bonferroni corrections were applied in all models. Violin plots were produced in R statistical software version 4.0.0 (R Project for Statistical Computing) using ggplot to provide a visualization of differences in *WISC-IV* index scores by sex and genetic status.

*P* values were 2-sided, and statistical significance was set at *P* < .05. Data were analyzed from July through November 2020.

## Results

### Demographic Characteristics

Among 1354 children (695 [51.3%] girls; mean [SD] age, 11.64 [2.64] years), there were 265 individuals with the *PSEN1* E280A variant (19.6%) and 1089 individuals without the variant (80.4%). Children with and without the variant did not differ by mean (SD) age (11.60 [2.71] years vs 11.64 [2.62] years; *P* = .81), mean (SD) years of education (5.35 [2.68] years vs 5.38 [2.66]; *P* = .89), urban living status (184 children [69.4%] vs 727 children [66.8%]; *P* = .41), SES (eg, low SES: 120 children [45.3%] vs 481 children [44.2%]; *P* = .20), or proportion by sex in each group (with variant: 124 [46.8%] boys vs 141 [53.2%] girls; without variant: 535 [49.1%] boys vs 554 [50.9%] girls; *P* =.50) (Table 1).

**Table 1.** Descriptive Statistics of Overall Sample

Variable	Children, No. (%) (N = 1354)		*P* value^a^
	With *PSEN1* variant (n = 265)	Without *PSEN1* variant (n = 1089)	
Sex			
Boys	124 (46.8)	535 (49.1)	.50
Girls	141 (53.2)	554 (50.9)	
Age, mean (SD) [range], y	11.60 (2.71) [6-16]	11.64 (2.62) [7-16]	.81
Years of education, mean (SD) [range]	5.35 (2.68) [0-12]	5.38 (2.66) [0-12]	.89
Urbanity			
Urban	184 (69.4)	727 (66.8)	.41
Rural	81 (30.6)	362 (33.2)	
SES			
Lower low	91 (34.3)	356 (32.7)	.20
Low	120 (45.3)	481 (44.2)	
Upper low	52 (19.6)	223 (20.5)	
Medium	0 (0)	24 (2.2)	
Medium high	1 (0.4)	4 (0.4)	
High	1 (0.4)	1 (0.1)	

Abbreviations: *PSEN1*, presenilin 1; SES, socioeconomic status.

^a^ *P* value is calculated using Welch 2 independent *t* test to compare groups by age and education and Pearson χ^2^ test to compare groups by sex, urbanity, and SES.

A subsample of all children with the variant and 192 children without the variant was examined to determine if performance on the *WISC-IV* indices differed between the groups when examining only participants who had at least 1 immediate parent with the variant. This subsample differed on urbanity, with more children with the variant living in urban areas than children without the variant, but not on other demographic variables (eTable 1 in the Supplement). Additionally, girls had statistically significantly more mean (SD) years of formal education than boys irrespective of genetic status in the full sample (5.64 [2.65] years vs 5.08 [2.66] years; *P* < .001) and subsample (5.76 [2.68] years vs 5.16 [2.70] years; *P* = .02) (eTable2 and eTable3 in the Supplement); sex differences were not statistically significant on demographic variables within genetic groups.

### Cognitive Performance

For the *WISC-IV* FSIQ, scores for all included participants were within 2 SDs of the assumed mean FSIQ value of 100. The range of included scores is 70 to 130. The overall mean FSIQ score was 87.91 (95% CI, 87.33-88.49).

#### Model 1: Genetic Status

There were no statistically significant differences in performance on *WISC-IV* indices by *PSEN1* E280A status after controlling for sex, urbanity, SES, and education in the full sample. For example, the mean scores for children with the variant vs those without the variant were 90.09 (95% CI, 86.68-91.51) vs 89.41 (95% CI, 88.72-90.11) (*P* = .40) for the VCI and 91.26 (95% CI, 89.95-92.58) vs 92.00 (95% CI, 91.35-92.65) (*P* = .32) for the WMI (Table 2). Additionally, there were no statistically significant differences in the sample of participants with a parent with the variant. For example, the mean scores for children with the variant vs those without the variant in this subsample were 90.00 (95% CI, 88.52-91.48) vs 91.53 (95% CI, 89.78-93.27) (*P* = .19) for the VCI and 91.20 (95% CI, 89.96-92.43) vs 92.81 (95% CI, 91.34-94.27) (*P* = .10) for the WMI (Table 3).

**Table 2.** Performance on *WISC-IV* Indices by Genetic Status in Overall Sample

*WISC-IV* index	Index standard score, mean (95% CI)^a^		*P* value^b^
	With *PSEN1* variant (n = 265)	Without *PSEN1* variant (n = 1089)	
Verbal comprehension	90.09 (86.68-91.51)	89.41 (88.72-90.11)	.40
Perpetual reasoning	92.70 (91.36-94.04)	92.13 (91.47-92.79)	.45
Working memory	91.26 (89.95-92.58)	92.00 (91.35-92.65)	.32
Processing speed	86.98 (85.60-88.36)	87.84 (87.16-88.52)	.27

Abbreviations: *PSEN1*, presenilin 1; *WISC-IV*, *Wechsler Intelligence Scale for Children, Fourth Edition*.

^a^ Data are reported as mean (95% CI) based on modified marginal means for the model with covariates and *P* value for the univariate general linear model.

^b^ Calculated using GLM with urbanity, education, and socioeconomic status entered as covariates and *PSEN1* genetic status, sex, and the interaction of genetic status and sex entered as fixed factors.

**Table 3.** Performance on *WISC-IV* Indices by Genetic Status Among Children With a Parent With Variant

*WISC-IV* index	*WISC-IV* index standard score, mean (95%CI)^a^		*P* value^b^
	With *PSEN1* variant (n = 265)	Without *PSEN1* variant (n = 192)	
Verbal comprehension	90.00 (88.52-91.48)	91.53 (89.78-93.27)	.19
Perpetual reasoning	92.60 (91.27-93.93)	92.40 (90.83-93.62)	.85
Working memory	91.20 (89.96-92.43)	92.81 (91.34-94.27)	.10
Processing speed	86.88 (85.49-88.26)	87.88 (86.25-89.51)	.36

Abbreviations: *PSEN1*, presenilin 1; *WISC-IV*, *Wechsler Intelligence Scale for Children, Fourth Edition*.

^a^ Data are reported as mean (95% CI) based on modified marginal means for the model with the covariates and *P* value for the univariate general linear model.

^b^ Calculated using general linear model with urbanity, education, and socioeconomic status entered as covariates and *PSEN1* genetic status, sex, and the interaction of genetic status and sex entered as fixed factors.

#### Model 2: Sex

Boys had statistically significantly decreased mean scores compared with girls on the WMI (90.27 [95% CI, 89.21-91.34] vs 92.99 [95% CI, 91.98-93.99]; mean difference = −2.72; *P* < .001), PRI (91.56 [95% CI, 90.47-92.65] vs. 93.27 [95% CI, 91.23-94.30]; mean difference = −1.71; *P* = .03), and VCI (88.69 [95% CI, 87.54-89.84] vs. 90.81 [95% CI, 89.73-91.90]; mean difference = −2.12; *P* = .009) (eTable 4 in the Supplement), irrespective of genetic status. In the sample of participants with a parent with the variant, boys had statistically significantly lower mean scores on the WMI (90.26 [95% CI, 88.92-91.60] vs. 93.75 [92.38-95.11]; mean difference = −3.49; *P* < .001) and PRI (91.37 [95% CI, 89.93-92.81] vs. 93.62 [92.15-95.09]; mean difference = −2.25; *P* = .03) (eTable 5 in the Supplement).

#### Model 3: Sex and Genetic Status Interaction

We found a statistically significant interaction between sex and genetic status, wherein boys with the variant in the full sample had worse mean WMI scores (88.78 [95% CI, 86.86-90.70]) than girls with the variant (93.75 [95% CI, 91.95-95.55]; mean difference = −4.97 [ie, approximately −0.33 SD difference in *WISC-IV* standard score]; *P* = .001), boys without the variant (91.77 [95% CI, 90.85-92.70]; mean difference = −2.99; *P* = .04), and girls without the variant (92.22 [95% CI, 91.32-93.13]; mean difference = −3.44; *P* = .009) (Table 4). The Figure provides visualizations of the distributions of the *WISC-IV* indices by sex and genetic status in the full sample. In the subsample, boys with the variant had statistically significantly worse mean WMI scores than girls with the variant (88.69 [95% CI, 86.87-90.50] vs. 93.71 [95% CI, 92.01-95.40]; mean difference = −5.02; *P* = .001) and girls without the variant (88.69 [95% CI, 86.87-90.50] vs. 93.79 [95% CI, 91.64-95.95]; mean difference = −5.10; *P* = .003) but not boys without the variant (eTable 6 in the Supplement).

**Table 4.** Performance on *WISC-IV* Indices by Sex and Genetic Status in Overall Sample

*WISC-IV* index	*WISC-IV* index standard score, mean (95% CI)^a^			
	With *PSEN1* variant		Without *PSEN1* variant	
	Boys (n = 124)	Girls (n = 141)	Boys (n = 535)	Girls (n =554)
Verbal comprehension	88.50 (86.44-90.57)	91.69 (89.75-93.62)	88.88 (87.89-89.88)	89.94 (88.97-90.92)
Perpetual reasoning	91.46 (89.50-93.42)	93.94 (92.10-95.77)	91.66 (90.71-92.60)	92.59 (91.68-93.52)
Working memory	88.78 (86.86-90.70)	93.75 (91.95-95.55)	91.77 (90.85-92.70)	92.22 (91.32-93.13)
*P* value^b^	NA	.001	.04	.009
Processing speed	85.77 (83.76-87.79)	88.18 (86.29-90.07)	88.10 (87.14-89.08)	87.58 (86.62-88.53)

Abbreviations: *PSEN1*, presenilin 1; *WISC-IV*, *Wechsler Intelligence Scale for Children, Fourth Edition*.

^a^ Data are reported as mean (95% CI) based on modified marginal means for the model with the covariates and *P* value for the univariate general linear model.

^b^ *P* values are calculated compared with boys with the *PSEN1* E280A variant using Bonferroni correction. Calculated using general linear model with urbanity, education, and socioeconomic status entered as covariates and *PSEN1* genetic status, sex, and the interaction of genetic status and sex entered as fixed factors.

**Figure.**
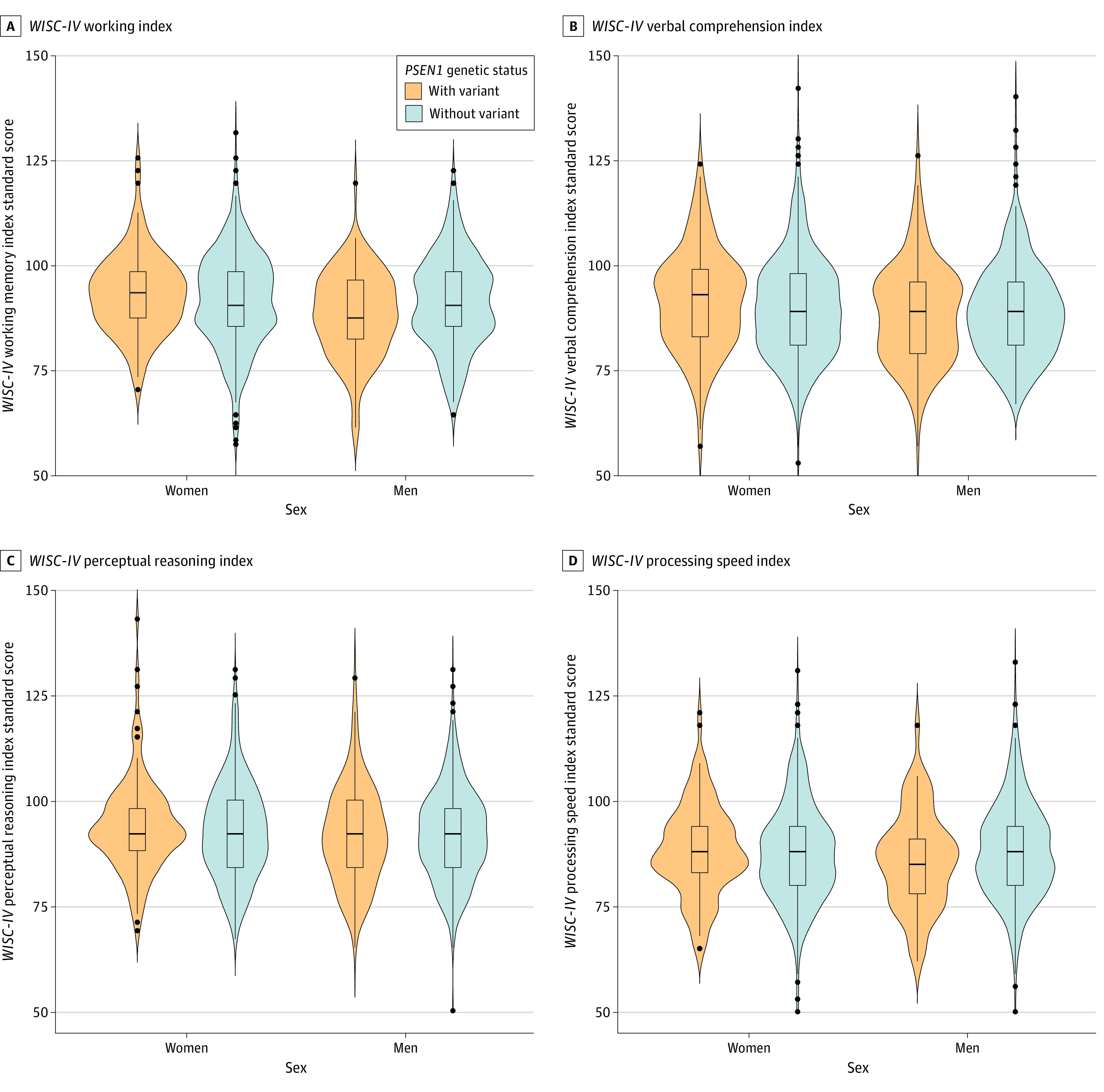
Performances on *WISC-IV* Indices by Sex and Genetic Status Shown are violin plots of standard score performances of children with the variant (in orange) and without the variant (in blue) on *WISC-IV* by biological sex. Thick middle lines of internal box plots indicate median values; tops and bottoms of boxes, interquartile range; whiskers, variability in scores outside of the interquartile values; black circles, statistical outliers; width of violin plot at any point, probability of values from the represented sample being at that value, with wider sections representing higher frequencies of values at that point in the distribution; *WISC-IV*, *Wechsler Intelligence Scale for Children, Fourth Edition*. Clinically, standard scores have a mean (SD) of 100 (15).

## Discussion

In this cohort study, we used cognitive data from a large sample to examine the cognitive functioning of *PSEN1* E280A ADAD in childhood. The *PSEN1* E280A variant is associated with the development of the AD clinical syndrome in midlife with virtually 100% certainty.^1^ Compared with children without this variant, children with the variant from this kindred (aged 6-16 years) were previously found to have increased gray matter volume in temporal and parietal brain regions, as well as decreases in deactivation in the parietal regions during an episodic memory task.^3^ In adults with the variant from this cohort, sex differences in verbal memory performances favored women,^4^ warranting the study of sex differences in cognition in childhood. Our study is novel, given that it is the first report, to our knowledge, on cognitive functioning among children with an ADAD variant. The homogeneity of the clinical course of this single-variant *PSEN1* E280A ADAD cohort allowed us to characterize the cognitive performance of children with the variant decades before the median age of onset of MCI for this variant (EYO −38 to −28 years for this sample).^2^

Contrary to our hypotheses, we found no differences in cognitive performance on the *WISC-IV* indices by *PSEN1* E280A variant status after controlling for urbanity, SES, and education. We found no differences in the entire sample, which included children without the variant who had an extended family member (eg, an uncle or aunt or a grandparent) with the ADAD variant, as well as in a subsample that included only participants who had a biological parent who also had the *PSEN1* E280A variant. When controlling for the same covariates, irrespective of genetic status, boys performed worse than girls in the full sample on the WMI, PRI, and VCI; in the subsample, boys performed worse than girls on the WMI and PRI. Additionally, we found a significant interaction between sex and genetic status in the full sample, wherein boys with the variant had worse WMI performance than girls with the variant, boys without the variant, and girls without the variant; in the subsample, boys with the variant performed worse on this index compared with girls with and without the variant. This partially supports our hypothesis that boys with the variant would exhibit weaker cognitive performance than girls with and without the variant, although we had anticipated to see significantly worse performances among boys with the variant compared with girls across all *WISC-IV* indices. The magnitude of the differences in WMI scores between boys with the variant and other groups was statistically significant but small from a clinical perspective. The largest difference of the adjusted means on this index between boys and girls corresponded to an approximately 5-point standard score difference (ie, an approximately −0.33 SD worse performance).

Although we found a clinically small difference, our findings are consistent with a 2020 study^4^ from our group that found that women with the variant had better verbal memory performance than men with the variant. In prototypical aging and sporadic AD, healthy older women have been found to have higher scores on verbal episodic memory tests compared with healthy older men.^5^ The literature from children without developmental impairments suggests that there may be differential brain networks recruited during working memory tasks by boys and girls.^15^ Additionally, the type of working memory task itself may be associated with sex differences seen in children, with boys who do not have developmental impairments performing better on tests of mental arithmetic and girls without developmental impairments performing better on tests of digit span.^9^ In this study, working memory performance on the *WISC-IV* was evaluated with 2 auditory tests (ie, digit span and letter-number sequencing),^7,8^ and we found that girls, irrespective of genetic status, had better WMI scores, aligning with prior meta-analytic research.^9^ Boys with the variant, however, appeared to be driving the at-large sex difference in this study, given that there were no significant differences in *WISC-IV* performance when examining boys and girls without the variant.

There are potential cognitive and academic implications of worse working memory performances compared with their peers among boys with ADAD variants. In children without developmental impairments, auditory working memory performance has been associated with verbal episodic memory performance.^16^ It is possible that early life differences in working memory are associated with sex differences seen in adulthood on episodic memory measures in this cohort.^4^ Auditory working memory was also found to be an important factor associated with reading comprehension,^17,18^ and worse working memory performance in childhood was associated with an increased likelihood of dropping out of secondary school.^19^ Decreased working memory performance in boys with variants needs to be further studied, as this increased risk could lead to downstream learning difficulties that may impact early life educational attainment. Future investigations should explore how this ADAD variant is specifically associated with acquiring academic skills (eg, reading speed and comprehension) and educational attainment (eg, number of school years repeated and frequency of school dropout), as well as the association of various sociodemographic factors with cognition among children with an ADAD variant. In this study, children with and without a variant did not differ on years of educational attainment, SES, or urbanity, but irrespective of genetic status, boys in this study attained approximately half a year less of formal education than their age-matched female peers. Enhancing early life educational experiences and quality has been suggested as a modifiable risk factor associated with dementia due to sporadic causes^20^ and is an important avenue of primary prevention research in ADAD. One study^21^ found that parental educational attainment can be associated with educational outcomes for children. Therefore, we need to explore possible interactions between parent education and cognitive and academic performance in individuals with and without the *PSEN1* variant, as well as the association of SES, educational quality, and growing up in urban vs rural environments with cognitive outcomes.^22,23^

### Limitations

This study has several limitations, including uncertainty regarding generalizability to children with other ADAD variants. The risk of bias in the unbalanced number of children with and without the variant in the sample must also be noted, given that theoretically the ratio should be closer to 1:1 given the autosomal dominant nature of the *PSEN1* E280A variant. The registry for this study, however, enrolls individuals without the variant who can trace their lineage to the extended *PSEN1* E280A cohort but who do not necessarily have a parent with the variant. This likely contributed, at least in part, to bias in the sample. Another limitation of this study is that the environmental stress associated with caring for a parent with cognitive decline due to ADAD may not be an experience shared equally by children with and without the variant. Some children without the variant in the large sample did not have a biological parent with the variant but did have an extended relative with the variant. Owing to the bias of the large sample and the possible outcomes associated with caring for a parent who is progressing to the AD clinical syndrome, we chose to also run our analyses in a subsample consisting of children with and without the variant who also had at least 1 parent with the variant, and this analysis yielded similar results to those of the entire sample.

Another limitation of this study is its cross-sectional nature, whereas childhood is an extended period of physical and cognitive development. As the onset of adolescence is variable, our future work will consider the value of collecting data on the age of puberty onset and examine its association with sex differences in cognitive performance among individuals with the *PSEN1* E280A variant. Some previous work has suggested that the early onset of menarche may be negatively associated with intelligence in the general population.^24^ Longitudinal studies are needed to identify the mechanism associated with these cognitive differences between boys and girls who have the variant and to determine the approximate age at which these differences emerge. Additionally, although participants were compared using the same *WISC-IV* norms from Mexico, *WISC-IV* norms for Colombia need to be developed given that children without the variant had lower index level performances than the expected mean value of 100 standard score seen in a reference sample.

## Conclusions

We characterized the cognitive performance of children with an ADAD variant compared with children without the variant from the same kindred cohort. Overall, children with the *PSEN1* E280A variant did not differ from children without the variant in performance across a wide set of cognitive domains. Boys with the variant, however, had decreased working memory performance compared with girls with and without the variant. These results may provide a foundation to investigate the earliest cognitive signs of ADAD and underscore the need for primary prevention research aimed at enhancing early life cognitive and educational experiences of ADAD cohort members.

## Notes

Supplement. **eTable 1.** Descriptive Statistics of 457 Children With a Parent With Variant **eTable 2.** Descriptive Statistics of 1354 Children in Full Sample by Sex **eTable 3.** Descriptive Statistics of 457 Children With a Parent With Variant by Sex **eTable 4.** Performance on *Wechsler Intelligence Scale for Children, Fourth Edition* Indices by Sex in Full Sample **eTable 5.** Performance on *Wechsler Intelligence Scale for Children, Fourth Edition* Indices by Sex When a Parent Has Variant **eTable 6.** Performance on *Wechsler Intelligence Scale for Children, Fourth Edition* Indices by Sex and Genetic Status When a Parent Has Variant
