# Supplementary material for: Sex Differences in Cognitive Abilities Among Children With the Autosomal Dominant Alzheimer Disease Presenilin 1 E280A Variant From a Colombian Cohort
Source: JAMA Netw Open. 2021 Aug 31;4(8):e2121697. doi: 10.1001/jamanetworkopen.2021.21697 (PMC8408665; doi:10.1001/jamanetworkopen.2021.21697)
Supplement: Supplement. — eTable 1. Descriptive Statistics of 457 Children With a Parent With Variant eTable 2. Descriptive Statistics of 1354 Children in Full Sample by Sex eTable 3. Descriptive Statistics of 457 Children With a Parent With Variant by Sex eTable 4. Performance on Wechsler Intelligence Scale for Children, Fourth Edition Indices by Sex in Full Sample eTable 5. Performance on Wechsler Intelligence Scale for Children, Fourth Edition Indices by Sex When a Parent Has Variant eTable 6. Performance on Wechsler Intelligence Scale for Children, Fourth Edition Indices by Sex and Genetic Status When a Parent Has Variant [file jamanetwopen-e2121697-s001.pdf]

Supplemental Online Content

Fox-Fuller JT, Artola A, Chen K, et al. Sex differences in cognitive abilities among children with the autosomal dominant Alzheimer disease presenilin 1 E280A variant from a Colombian cohort. *JAMA Netw Open*. 2021;4(8):e2121697. doi:10.1001/jamanetworkopen.2021.21697

eTable 1. Descriptive Statistics of 457 Children With a Parent With Variant

eTable 2. Descriptive Statistics of 1354 Children in Full Sample by Sex

eTable 3. Descriptive Statistics of 457 Children With a Parent With Variant by Sex

eTable 4. Performance on *Wechsler Intelligence Scale for Children, Fourth Edition* Indices by Sex in Full Sample

eTable 5. Performance on *Wechsler Intelligence Scale for Children, Fourth Edition* Indices by Sex When a Parent Has Variant

eTable 6. Performance on *Wechsler Intelligence Scale for Children, Fourth Edition* Indices by Sex and Genetic Status When a Parent Has Variant

This supplemental material has been provided by the authors to give readers additional information about their work.

© 2021 Fox-Fuller JT et al. *JAMA Network Open.*

eTable 1. Descriptive Statistics of 457 Children With a Parent With Variant

	PSEN1		
**Variable**	**Carriers, Mean, n=265**	**Non-Carriers, Mean, n=192**	***p*-value**
**Sex (%)**			
Male	124 (46.4%)	104 (54.2%)	0.12
Female	141 (53.6%)	88 (45.8%)	
**Age in years (SD)**			
Mean<br>Range	11.60 (2.71)<br>6-16	12.02 (2.71)<br>8-16	0.10
**Years of education (SD)**			
Mean<br>Range	5.35 (2.68)<br>0-12	5.62 (2.73)<br>0-12	0.30
**Urbanity (%)**<br>Urban<br>Rural	 184 (69.4%)<br>81 (30.6%)	 112 (58.3%)<br>80 (41.7%)	 0.01*

© 2021 Fox-Fuller JT et al. *JAMA Network Open.*

**SES (%)**			
Lower-low	91 (34.3%)	60 (31.3%)	0.48
Low	120 (45.3%)	100 (52.1%)	
Upper-low	52 (19.6%)	30 (15.6%)	
Medium	0 (0%)	1 (0.5%)	
Medium-high	1 (0.4%)	0 (0.5%)	
High	1 (0.4%)	1 (0.5%)	

Abbreviations: PSEN1 =: Presenilin 1; SD = Standard deviation; SES =Socioeconomic status.

Note: P-value is calculated using Welch's two independent t-test to compare groups by age and education and Pearson's chi-squared test to compare groups by sex, urbanity, and SES.

p-values are calculated relative to males (Bonferroni correction)
**p*<.05

© 2021 Fox-Fuller JT et al. *JAMA Network Open.*

eTable 2. Descriptive Statistics of 1354 Children in Full Sample by Sex

**Variable**	**Males, n=659**	**Females, n=695**	***p*-value**
**Carrier status (%)**			
PSEN1 carrier	124 (54.4%)	141 (61.6%)	0.50
Non-carrier	535 (45.6%)	554 (38.4%)	
**Age in years (SD)**			
Mean	11.63 (2.66)	11.64 (2.62)	0.95
Range	6-16	7-16	
**Years of education (SD)**			
Mean	5.08 (2.66)	5.64 (2.65)	<.001***
Range	0-12	1-12	
**Urbanity (%)**			
Urban	450 (68.3%)	461 (66.3%)	0.44
Rural	209 (31.7%)	234 (33.7%)	
**SES (%)**			

© 2021 Fox-Fuller JT et al. *JAMA Network Open.*

Lower-low<br>Low<br>Upper-low<br>Medium<br>Medium-high<br>High	210 (31.9%)<br>288 (43.7%)<br>141 (21.4%)<br>16 (2.4%)<br>2 (0.3%)<br>2 (0.3%)	237 (34.1%)<br>313 (45.0%)<br>134 (19.3%)<br>8 (1.2%)<br>3 (0.4%)<br>0 (0%)	0.24

Abbreviations: PSEN1 =: Presenilin 1; SD = Standard deviation; SES =Socioeconomic status.

Note: P-value is calculated using Welch's two independent t-test to compare groups by age and education and Pearson's chi-squared test to compare groups by carrier status, urbanity, and SES.

*p<.05
**p<.01
***p<.001

© 2021 Fox-Fuller JT et al. *JAMA Network Open.*

eTable 3. Descriptive Statistics of 457 Children With a Parent With Variant by Sex

Variable	Males, n=228	Females, n=229	*p*-value
**Carrier status (%)**			
PSEN1 carrier	124 (54.4%)	141 (61.6%)	0.12
Non-carrier	104 (45.6%)	88 (38.4%)	
**Age in years (SD)**			
Mean	11.78 (2.71)	11.78 (2.72)	0.99
Range	6-16	8-16	
**Years of education (SD)**			
Mean	5.16 (2.70)	5.76 (2.68)	0.02*
Range	0-12	1-12	
**Urbanity (%)**			
Urban	150 (65.8%)	146 (63.8%)	0.65
Rural	78 (34.2%)	83 (36.2%)	
**SES (%)**			

© 2021 Fox-Fuller JT et al. *JAMA Network Open.*

Lower-low<br>Low<br>Upper-low<br>Medium<br>Medium-high<br>High	71 (31.2%)<br>110 (48.2%)<br>43 (18.9%)<br>1 (0.4%)<br>1 (0.4%)<br>2 (0.9%)	80 (34.9%)<br>110 (48.1%)<br>39 (17.0%)<br>0 (0%)<br>0 (0%)<br>0 (0%)	0.45

Abbreviations: PSEN1 =: Presenilin 1; SD = Standard deviation; SES =Socioeconomic status.

Note: P-value is calculated using Welch's two independent t-test to compare groups by age and education and Pearson's chi-squared test to compare groups by carrier status, urbanity, and SES.

*p<.05

© 2021 Fox-Fuller JT et al. *JAMA Network Open.*

eTable 4. Performance on *Wechsler Intelligence Scale for Children, Fourth Edition* Indices by Sex in Full Sample

WISC-IV Index	Males WISC-IV Index Standard Score, Mean (95% CI), n=659	Females WISC-IV Index Standard Score, Mean (95% CI), n=695
Verbal Comprehension	**88.69 (87.54-89.84)**	90.81 (89.73-91.90), *p*=.009**
Perpetual Reasoning	**91.56 (90.47-92.65)**	93.27 (92.23-94.30), *p*=.03*
Working Memory	**90.27 (89.21-91.34)**	92.99 (91.98-93.99), *p*<.001***
Processing Speed	86.94 (85.20-88.06)	87.88 (86.82-88.94)

p-values are calculated relative to males (Bonferroni correction)
**p*<.05
***p*<.01
****p*<.001

Abbreviations: WISC-IV = Wechsler Intelligence Scale for Children, Fourth Edition; CI = Confidence Interval
Note: Data are reported as the mean (95% CI) based on modified marginal means for the model with the co-variates, p-value for the univariate general linear model (GLM).

[a] Calculated using GLM with urbanity, education, and socioeconomic status entered as covariates, and PSEN1 carrier status, sex, and the interaction of carrier status and sex entered as fixed factors.

© 2021 Fox-Fuller JT et al. *JAMA Network Open.*

eTable 5. Performance on *Wechsler Intelligence Scale for Children, Fourth Edition* Indices by Sex When a Parent Has Variant

WISC-IV Index	Males WISC-IV Index Standard Score, Mean (95% CI), n=228	Females WISC-IV Index Standard Score, Mean (95% CI), n=229
Verbal Comprehension	89.84 (88.24-91.44)	91.69 (90.05-93.32)
Perpetual Reasoning	**91.37 (89.93-92.81)**	93.62 (92.15-95.09), *p*=.03*
Working Memory	**90.26 (88.92-91.60)**	93.75 (92.38-95.11), *p*<.001***
Processing Speed	86.72 (85.22-88.20)	88.04 (86.51-89.57)

p-values are calculated relative to males (Bonferroni correction)
*$p<.05$
**$p<.01$
***$p<.001$

Abbreviations: WISC-IV = Wechsler Intelligence Scale for Children, Fourth Edition; CI = Confidence Interval; PSEN=Preseinlin1
Note: Data are reported as the mean (95% CI) based on modified marginal means for the model with the co-variates, p-value for the univariate general linear model (GLM).
[a] *Calculated using GLM with urbanity, education, and socioeconomic status entered as covariates, and PSEN1 carrier status, sex, and the interaction of carrier status and sex entered as fixed factors.*

© 2021 Fox-Fuller JT et al. *JAMA Network Open.*

eTable 6. Performance on *Wechsler Intelligence Scale for Children, Fourth Edition* Indices by Sex and Genetic Status When a Parent Has Variant

**WISC-IV Index**	***PSEN1*** **Male Carriers, WISC-IV Index Standard Score, Mean (95% CI), n=124**	***PSEN1*** **Female Carriers, WISC-IV Index Standard Score, Mean (95% CI), n=141**	**Male Non-Carriers WISC-IV Index Standard Score, Mean (95% CI), n=535**	**Female Non-Carriers, WISC-IV Index Standard Score, Mean (95% CI), n=554**
Verbal Comprehension	88.51 (86.34-90.68)	91.48 (89.46-93.51)	91.16 (88.81-93.52)	91.89 (89.32-94.45)
Perpetual Reasoning	91.30 (89.35-93.26)	93.89 (92.07-95.71)	91.44 (89.32-93.55)	93.35 (91.05-95.66)
Working Memory	**88.69 (86.87-90.50)**	93.71 (92.01-95.40), *p*=.001**	91.83 (89.86-93.80)	93.79 (91.64-95.93), *p*=.003**
Processing Speed	85.53 (83.50-87.56)	88.22 (86.33-90.12)	87.91 (85.71-90.11)	87.85 (85.45-90.26)

p-values are calculated relative to male *PSEN1* E280A carriers with Bonferroni correction
***p*<.01

Abbreviations: WISC-IV = Wechsler Intelligence Scale for Children, Fourth Edition; PSEN1 = Presenilin1; CI = Confidence Interval

Note: Data are reported as the mean (95% CI) based on modified marginal means for the model with the co-variates, p-value for the univariate general linear model (GLM).

[a] *Calculated using GLM with urbanity, education, and socioeconomic status entered as covariates, and PSEN1 carrier status, sex, and the interaction of carrier status and sex entered as fixed factors.*

© 2021 Fox-Fuller JT et al. *JAMA Network Open.*
